# Malaria prevalence, severity and treatment outcome in relation to day 7 lumefantrine plasma concentration in pregnant women

**DOI:** 10.1186/s12936-016-1327-1

**Published:** 2016-05-13

**Authors:** Ritah F. Mutagonda, Appolinary A. R. Kamuhabwa, Omary M. S. Minzi, Siriel N. Massawe, Betty A. Maganda, Eleni Aklillu

**Affiliations:** Department of Clinical Pharmacy and Pharmacology, School of Pharmacy, Muhimbili University of Health and Allied Sciences, P.O. BOX 65013, Dar es Salaam, Tanzania; Department of Obstetrics and Gynaecology, School of Medicine, Muhimbili University of Allied Sciences, P.O. BOX 65013, Dar es Salaam, Tanzania; Department of Laboratory of Medicine, Division of Clinical Pharmacology, Karolinska Institutet, 141 86 Stockholm, Sweden

**Keywords:** Malaria, Pregnancy, Lumefantrine, Recurrent parasitaemia, Tanzania

## Abstract

**Background:**

Day 7 plasma concentrations of lumefantrine (LF) can serve as a marker to predict malaria treatment outcome in different study populations. Two main cut-off points (175 and 280 ng/ml) are used to indicate plasma concentrations of LF, below which treatment failure is anticipated. However, there is limited data on the cumulative risk of recurrent parasitaemia (RP) in relation to day 7 LF plasma concentrations in pregnant women. This study describes the prevalence, severity, factors influencing treatment outcome of malaria in pregnancy and day 7 LF plasma concentration therapeutic cut-off points that predicts treatment outcome in pregnant women.

**Methods:**

This was a one-arm prospective cohort study whereby 89 pregnant women with uncomplicated *Plasmodium falciparum* malaria receiving artemether-lumefantrine (ALu) participated in pharmacokinetics and pharmacodynamics study. Blood samples were collected on days 0, 2, 7, 14, 21 and 28 for malaria parasite quantification. LF plasma concentrations were determined on day 7. The primary outcome measure was an adequate clinical and parasitological response (ACPR) after treatment with ALu.

**Results:**

The prevalence of malaria in pregnant women was 8.1 % (95 % CI 6.85–9.35) of whom 3.4 % (95 % CI 1.49–8.51) had severe malaria. The overall PCR-uncorrected treatment failure rate was 11.7 % (95 % CI 0.54–13.46 %). Low baseline hemoglobin (<10 g/dl) and day 7 LF concentration <600 ng/ml were significant predictors of RP. The median day 7 LF concentration was significantly lower in pregnant women with RP (270 ng/ml) than those with ACPR (705 ng/ml) (p = 0.016). The relative risk of RP was 4.8 folds higher (p = 0.034) when cut-off of <280 ng/ml was compared to ≥280 ng/ml and 7.8-folds higher (p = 0.022) when cut-off of <600 ng/ml was compared to ≥600 ng/ml. The cut-off value of 175 ng/ml was not associated with the risk of RP (p = 0.399).

**Conclusions:**

Pregnant women with day 7 LF concentration <600 ng/ml are at high risk of RP than those with ≥600 ng/ml. To achieve effective therapeutic outcome, higher day 7 venous plasma LF concentration ≥600 ng/ml is required for pregnant patients than the previously suggested cut-off value of 175 or 280 ng/ml for non-pregnant adult patients.

## Background

Over 93 % of the Tanzania mainland population lives in areas where malaria is endemic. In Tanzania, there is great variation in the risk of malaria transmission and prevalence ranging from 1–33 %, with an average of about 10 % [1]. The levels of transmission are high in Lake zone regions, coastal regions and southern lowlands [2]. Despite declining of malaria prevalence in the country, recent reports show that malaria cases range between 10–12 million annually and is still a leading cause of outpatient, inpatient and hospital deaths with 60–80 thousands estimated death per year [2]. Pregnant women and children under 5 years of age are at high risk for malaria infections. The risk of malaria infection in pregnant women increases due to changes in hormonal levels and immune system [3]. In high-transmission settings, the adverse effects of *Plasmodium falciparum* infection in pregnancy are most pronounced for women in their first pregnancy whereas in low transmission settings malaria affects all pregnant women, regardless of the number of times they have been pregnant [4]. It is estimated that 1.7 million pregnant women in Tanzania mainland are susceptible to malaria infection per year. The overall prevalence of malaria parasitaemia among pregnant women residing on Lake zone regions was 12.2 % (95 % CI 11.5–12.8) [5].

Artemisinin-based combination therapy is recommended by WHO as first-line treatment for uncomplicated *P. falciparum* malaria in the second and third trimester of pregnancy [6]. The fixed dose drug combination of artemether and lumefantrine (ALu) is the first-line drug of choice to treat malaria in Tanzania [7]. Artemether is short-acting and has a very rapid and potent anti-malarial effect, resulting into prompt resolution of symptoms. The drug has a short half-life (1–3 h) and is rapidly metabolized into an active metabolite—dihydroartemisinin. Artemether and its active metabolite have been estimated to reduce parasite biomass by approximately 10,000-fold per reproductive cycle (every 2 days) [8]. Lumefantrine (LF) is long-acting and has longer elimination half-life killing nearly all residual parasites. Combining short and long acting drugs ensures clearance of all *P. falciparum* parasites and prevents recrudescence, thereby ensuring malaria cure [9].

Plasma LF concentrations at day 7 reflects the degree to which the residual parasites are exposed, and is generally considered as a useful pharmacokinetic marker to predict malaria treatment outcome [10, 11]. Different ‘therapeutic’ cut-off points of day 7 LF concentrations have been suggested ranging from 170–600 ng/ml below which treatment failure is anticipated for different study populations [10–18]. Therapeutic cut-offs points of 175 ng/ml [11], and 280 ng/ml [19] are the most commonly used. The relative risk of recrudescent malaria has been reported to be substantially higher in patients with day 7 concentrations at the cut-off point <175 ng/ml compared to those with higher concentration [11]. Day 7 plasma LF concentration of <280 ng/ml in Thai patients resulted in 51 % cure rate compared to 75 % cure rates in patients with concentration >280 ng/ml [19]. A recent pharmacokinetics study of mefloquine, piperaquine and ALu in Cambodia and Tanzania reported that the targeted day 7 LF concentration (>600 ng/ml) was not achieved in 100 (71 %)—non-pregnant adult patients [20]. The study reported that in Tanzania, 35 % of samples obtained from non-pregnant population had LF concentration below the cut off value of <175 ng/ml at day 7 [20]. Pregnancy is known to lower blood concentrations of artemether and LF, thus putting pregnant women at risk of under-dosing [20–22].

Malaria during pregnancy is associated with high maternal and perinatal mortality [6]. Pregnancy is an important factor affecting the pharmacokinetics of a number of drugs including anti-malarial drugs mainly due to reduced drug absorption, elevated drug metabolism, and rapid clearance rate and altered volume of distribution [23]. Activity of CYP3A4, the main enzyme responsible for LF metabolism, is increased during pregnancy [24]. Studies have reported lower venous plasma LF concentrations at day 7 in pregnant women compared with those in non-pregnant women [23, 25, 26]. Large proportion (30–40 %) of pregnant patients displayed day 7 plasma LF concentration below 280 ng/mL [13, 20, 22]. Sub-therapeutic plasma drug exposures may select for parasites with reduced drug susceptibility and increases the risk for development of drug resistance. Indeed, a higher treatment failure rate has been observed for pregnant women compared to non-pregnant women living in the same area [13, 22, 23, 27].

It is well recognized that plasma LF concentration on day 7 LF levels is a surrogate marker to predict treatment outcome, but the therapeutic cut of point may vary between different patient populations and transmission areas (low versus high). The therapeutic day 7 LF concentration threshold below which RP is anticipated needs to be defined better, particularly for high risk group living in malaria endemic countries [14], including pregnant women whose pharmacokinetic profile is altered due to pregnancy associated hormonal and physiological changes [28, 29]. Therefore, the aim of this study was to describe (i) malaria prevalence, severity and treatment outcome in pregnancy, and (ii) to describe the day 7 LF plasma concentration profile and identify its optimal therapeutic cut-off point which predicts malaria treatment outcome in pregnant women.

## Methods

### Study design and procedures

This was a one-arm prospective cohort study that included all pregnant women who gave consent to be screened for malaria when attending antenatal clinics [30] at Kisarawe and Mkuranga district hospitals, northern Tanzania. In this region transmission of malaria is perennial, which peaks by the end of the long and short rains from May to July and December to January, respectively. The prevalence of asexual parasitaemia in this area is 14 %, and *P. falciparum* is the predominant species [31].

The study received ethics approval from the institutional review board of Muhimbili University of Health and Allied Sciences (MUHAS). Participants were informed about the aim of the study and gave written consent before participating in the study. Confidentiality was ensured to all individuals who participated in the study, whereby, all collected samples and the filled confidential report forms (CRF) have a coded identification number.

### Sample size

The study aimed to obtain a sample size of 50, which is the minimum recommended by the WHO regardless of rates of failure anticipated, in order to be representative [32]. This is the first observational study to determine the prevalence of malaria in pregnant women from Tanzania and could only include malaria cases available by screening all pregnant women attending antenatal care at the study sites. Considering anticipated population proportion of clinical failures in pregnant women (P) being 18 % [23], with 95 % confidence level and 10 % precision, a minimum sample size of 50 would be needed. Adding a 20 % loss to follow the required sample size was 59 malaria positive pregnant women.

### Patients recruitment

Between May 2014 to April 2015, 1835 pregnant women attending the antenatal cares (ANCs) were screened by using malaria rapid diagnostic test (MRDT). Pregnant women with MRDT positive results were enrolled in the study. Inclusion criteria included women aged 18 years and above, resident of Mkuranga or Kisarawe districts, in the second or third trimester, with uncomplicated *P. falciparum* infection and haemoglobin level of >8 g/dl. Full medical history, including current illnesses and medication used, were recorded. Clinical examination on the day of enrollment and during follow-up visits on days 2, 7, 14 and 28 were done including axillary temperature measurement and malaria-related symptoms evaluation.

### Screening of pregnant women for malaria

Malaria was tested by using SD BIOLINE Malaria Ag P.f/Pan^®^ MRDT (Standard Diagnostics, Inc, Korea). The blood samples were collected by finger prick. About 5 μl of whole blood were added into the ‘sample well’ of respective test devices using a micropipette supplied with the test kit. Four drops of assay diluent were added into the ‘sample diluent well’. All the test results were recorded within 30 min. The sample was positive when there was appearance of a control line and a test line on the result window but it was negative when there was only a control line.

### Drug regimen

Enrolled study participants received four tablets of ALu (Coartem; Novartis Pharma AG, Basel, Switzerland) (20 mg artemether and 120 mg lumefantrine) over the course of 3 days at 0, 8, 24, 36, 48, and 60 h. In order to enhance absorption of ALu and standardize dose administration, patients were supplied with packets of milk containing 4.5 g of fat [33] and were instructed to swallow ALu tablets with 200 mls of milk. Patients were directed on how to take the remaining five doses of the drug at home and were asked to come to the hospital to take the last dose. Patients with microscopically-confirmed *P. falciparum* within 28 days of follow-up were treated with either artesunate or artemether or quinine injection as per the national malaria treatment guidelines [5] since this was presumptively regarded as treatment failure.

### Microscopy and haemoglobin determination

To estimate the parasite density, capillary blood from a finger prick was taken at days 0, 2, 7, 14, 21 and 28. Samples were collected on slides, Giemsa-stained thick smears were examined by two different experienced microscopists using light microscope. Plasmodium parasites were counted against 200 white blood cells (WBC) on the thick film. Five hundred WBCs were counted where less than ten parasites were observed. The parasite count was then multiplied by a factor of 40 or 16 depending on the counted WBCs. Haemoglobin was measured by HemoCue Hb 201 + ^®^ machine (HemoCue AB Ängelholm, Sweden) following manufacturers’ instructions.

### Quantification of plasma LF concentrations

In total, three millilitres of venous blood were drawn from the patients at random times on days 0 and 7 to determine plasma LF concentrations. Day 0 blood sample was collected before starting the medication as a baseline in order to determine the presence of LF in the patient’s plasma prior to treatment [34, 35]. Blood samples in heparinized vacutainer tubes were centrifuged at 2000 *g* for 5 min, and the plasma samples were stored in cryotubes. Samples were stored at −80 °C at MUHAS before analysis. Plasma LF concentration was analysed using a validated high performance liquid chromatography (HPLC) with ultraviolet detection at Sida/MUHAS bioanalytical laboratory in Dar-es-Salaam, Tanzania as described previously [36]. The coefficients of variation (CV %) during the analysis of LF were 8.4, 4.7 and 4.5 % at 100, 1000, and 8000 ng/ml, respectively. The lower limit of quantification was 50 ng/ml.

### Malaria treatment outcome

Treatment outcome classification was based on WHO recommendations on the methods for surveillance of anti-malarial drug efficacy as early treatment failure (ETF), late clinical failure (LCF), late parasitological failure (LPF), and Adequate clinical and parasitological response (ACPR) [32]. An ACPR in patients at day 28 after anti-malarial drug treatment was the primary study objective. The time to recurrent parasitaemia (RP) or the risk of RP was defined as the number of days between taking the first dose of ALu and the day of microscopically detecting malaria parasites in the thick blood film. The time at risk ended whenever one of the following conditions occurred: RP, loss to follow-up, withdrawal, or end of follow-up period [37].

### Data analysis

The data were analysed using the statistical package for social sciences (SPSS—IBM Corporation, Somers, NY) software, version 20. The intention-to-treat approach was used to analyse the anti-malarial treatment response. All day 7 LF plasma concentrations were first transformed into log 10 values before applying parametric tests. The relation of the outcome variable (treatment outcome) and explanatory variables were tested using independent *t* test (log plasma LF day 7 concentrations, age of the patient, gestational age, gravidity and baseline parasite count). The cumulative risk of RP was estimated using the Kaplan–Meier product limit formula and data were censored. A P value <0.05 was considered statistically significant.

## Results

### Study population characteristics

Baseline characteristics of all enrolled patients are presented in Table 1. The mean age of pregnant patients was 24.96 ± 6 (range 16–38) years whereas the majority (44 %) were multigravidae and in the second trimester (68.6 %). The median parasite density of the enrolled patients was 1400 (range 400–58,416) parasites/µl.

**Table 1:** Baseline characteristics of patients with *P. falciparum* malaria sampled for LF pharmacokinetics (n = 60)

Characteristic	Number of pregnant women	Percentage
Age (years)		
<20	16	*26.7*
25–35	38	*63.3*
>35	6	*10.0*
Gravida		
Primigravida	21	*35*
Secundigravida	13	*21.7*
Multigravida	26	*43.3*
Trimester		
Second	40	*66.7*
Third	20	*33.3*
Parasitaemia		
<1000	20	*33.3*
1000–10,000	33	*55.0*
>10,000	7	*11.7*

### Malaria prevalence and its severity in pregnancy

One thousand eight hundred and thirty five pregnant women were screened using MRDT during the study period and out of these 148 were positive for malaria. Prevalence of malaria in pregnancy during the study period was 8.1 % (95 % CI 6.85–9.35). Out of these malaria positive pregnant women, 5 (3.4, 95 % CI 1.49–8.51) had severe malaria and the rest (143 pregnant women) had uncomplicated *P. falciparum* infection. Severe malaria was prominent in primigravidae (four women) than multigravid pregnant women (one woman). MRDT positive samples were further analysed using microscope whereby, 111 out of 148 (75 %) samples were confirmed to have *P. falciparum* infection.

**Fig. 1:**
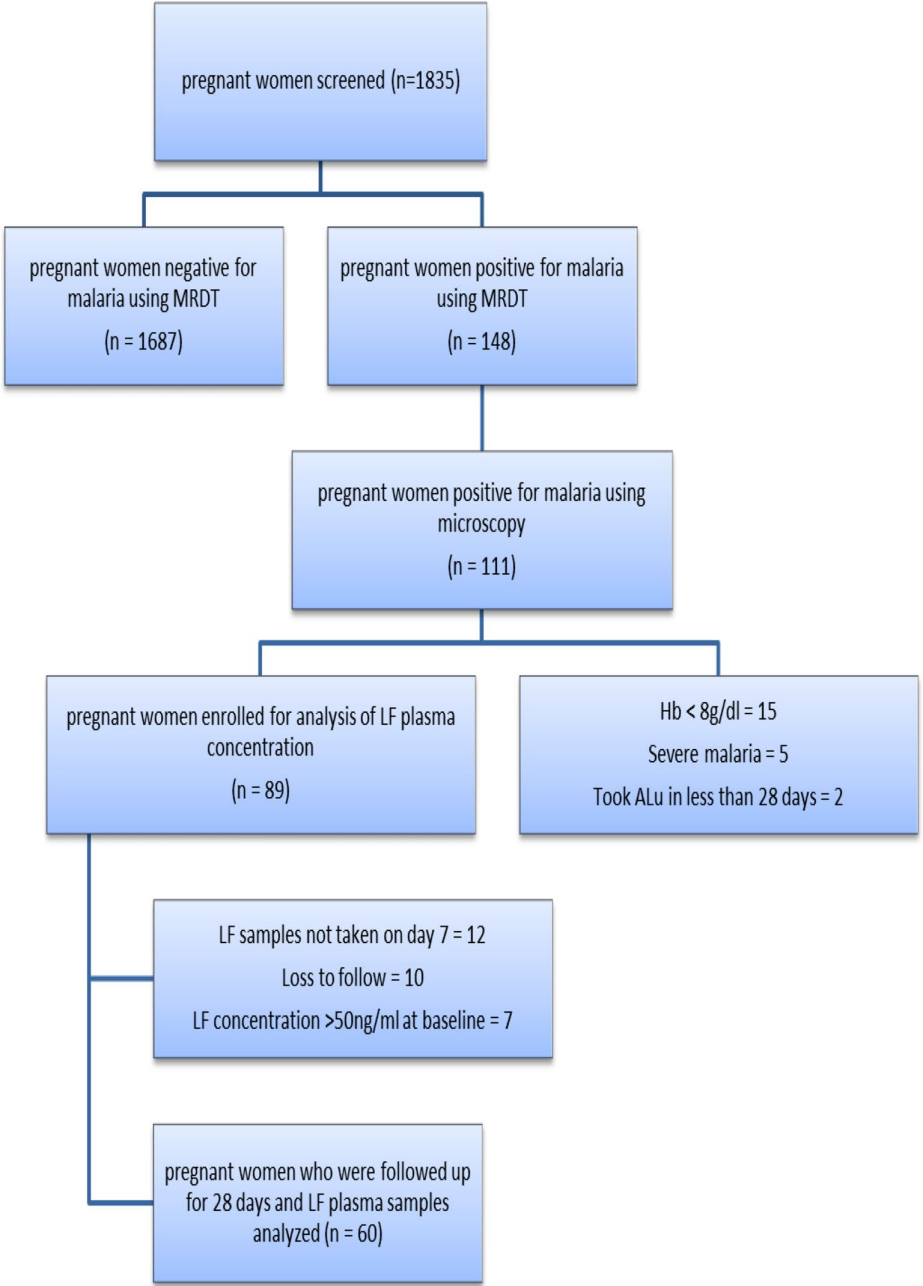
Patient recruitment flow chart for the malaria in pregnancy study in Kisarawe and Mkuranga Districts

Eighty-nine patients with uncomplicated *P. falciparum* malaria consented and were enrolled in this study. Flowchart for study enrolment is presented in Fig. 1. In this study, seven (7.9 %) of the enrolled pregnant women were excluded from the data analysis due to detection of plasma LF concentration (>50 ng/ml) at baseline, and twelve (13.5 %) patients did not return to the ANCs to provide blood samples for determination of day 7 LF concentration. Therefore, sixty pregnant women were available to provide blood samples for the whole period of 28 days follow up.

### Malaria treatment outcome in pregnancy

There were a total of seven (11.7; 95 % CI 0.54–13.46 %) therapeutic failures in this study among pregnant women. No ETF or LCF was observed in this study; nonetheless, seven pregnant women had LPF. Hence, the overall ACPR after 28 days of follow up was 88.3 % (95 % CI 40.37–65.63 %).

**Fig. 2:**
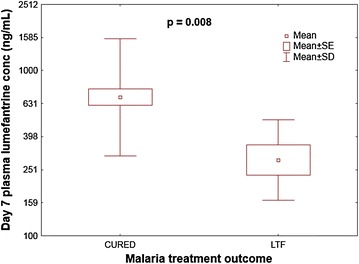
Comparison of day 7 plasma LF concentration in pregnant women with adequate clinical and parasitological response (CURED, *n* = *54*) versus those with late treatment failure (LTF, *n* = *7*) after treatment with ALu by day 28 using independent t test. Data was converted to log 10 value before analysis

Day 7 LF concentration was significantly lower among pregnant women with RP than those with ACPR (p = 0.016). The median plasma LF concentration among pregnant women with ACPR was 705 ng/ml (140–3059 ng/ml), whereas for women with LPF, it was 270 ng/ml (123.6–602 ng/ml) (Fig. 2). There was highly significant association between baseline Hb levels (p = 0.003) and day 7 LF plasma concentrations (p = 0.008) with RP. Other covariates, such as age, gravida, gestational age and baseline parasitaemia were not associated with RP (Table 2).

**Table 2:** Analysis of predictors of malaria treatment outcome in pregnant women

Characteristic	Mean (95 % CI)	P value
Age (years)		
RP	25.7 (21.3–30.1)	0.889
ACPR	25.36 (23.57–27.14)	
Gravida		
RP	2 (1–3)	0.529
ACPR	3 (2–3)	
Gestation age		
RP	22 (19–24)	0.714
ACPR	23 (21–24)	
Baseline parasitaemia		
RP	3960 (1464.4–9384.4)	0.502
ACPR	2688 (1443.0–3933.6)	
Baseline Hb (g/dl)		
RP	8.87 (8.11–9.64)	0.003
ACPR	10.33 (10.00–10.67)	
Day 7 LF levels (ng/ml)		
RP	2.46 (2.23–2.68)	0.008
ACPR	2.84 (2.74–2.94)	

Pregnant women with Hb <10 g/dl had 6.4-folds risk of RP than those with >10 g/dl whereas those with day 7 LF concentration <600 ng/ml had a 7.8-folds risk of RP than those with >600 ng/ml.

### Day 7 LF plasma concentrations and malaria treatment outcome

**Fig. 3:**
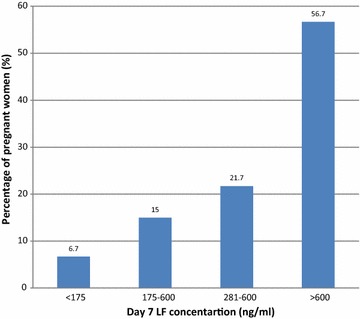
Day 7 LF concentrations for pregnant women treated for malaria (n = 60)

Day 7 median plasma concentrations of LF after administration of a six-dose regimen over three days were 650 ng/mL (Inter quartile range = 294–1195 ng/mL) for pregnant women. There was marked inter-individual variability in day 7 plasma LF concentrations (range = 123–3059 ng/mL, coefficient of variation = 81.1 %). There was no association between day 7 LF plasma concentrations with age (p = 0.784), gravida (p = 0.314), trimester (p = 0.496) or baseline parasitaemia (p = 0.644). Amongst of the studied population 6.7 % of the patients had day 7 concentrations below the therapeutic cut-off of 175 ng/ml, 21.7 % below 280 ng/ml, and 43.3 % below 600 ng/ml. More than half of the pregnant women (57 %) had day 7 concentration of >600 ng/ml which is required for maximal efficacy. Equally, 15 % had 175–280 ng/ml, and 21.7 % had 281–600 ng/ml (Fig. 3). Treatment outcome in relation to day 7 LF plasma levels in pregnant women was such that ACPR was 75, 66.7, 76.9 and 97.1 % when day 7 LF plasma concentrations were <175, <280, <600 and >600 ng/ml, respectively.

Independent t-test indicated highly significant association between high log day 7 plasma LF concentrations and ACPR (p = 0.008). The relative risk of RP was 2.3 (when comparing LF concentrations <175 to >175 ng/ml), 4.8 (when comparing LF concentrations <280 to >280 ng/ml) and 7.8-folds higher when comparing LF concentrations < 600 to >600 ng/ml. The relative risk of RP in patients at the cut-off point LF <175 ng/ml was not significantly higher than in those with LF concentration of >175 ng/ml (p = 0.399). On the other hand, relative risk of RP was statistically significant using the LF cut-off point of <280 ng/ml compared to >280 ng/ml (p = 0.034) and <600 ng/ml compared to >600 ng/ml (p = 0.022) (Table 3).

**Table 3:** Comparison of relative risk of RP in relation to day 7 LF cut-off plasma concentration in pregnant women

Day 7 LF cut-off conc. (ng/ml)	RR	95 % CI	P value (Fischer’s exact test)
<175 to >175	2.3	0.36–14.96	0.399
<280 to >280	4.8	1.23–18.88	0.034
<600 to >600	7.8	1.00–61.22	0.022

**Fig. 4:**
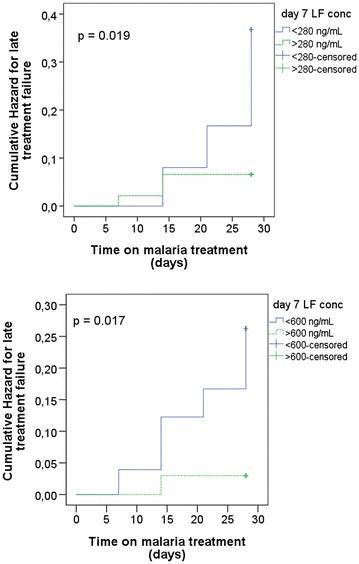
Kaplan Meier curve showing cumulative risk of RP in pregnant women with cut-off day 7 LF plasma concentration of <280 ng/mL compared to >280 ng/mL (*Top*) versus <600 ng/mL compared to >600 ng/mL (*below*). P value is from log rank test

Kaplan–Meier analysis of the PCR uncorrected data following treatment of uncomplicated malaria in pregnant women showed that there was no cumulative risk of RP regardless of day 7 LF concentration until on day 7 after the administration of the first dose of ALu. When the cut-off value was 280 ng/ml, the cumulative risk of RP was slightly increased on day 7 in patients with LF concentration >280 ng/ml than those with <280 ng/ml up to day 14. After that, the cumulative risk of RP was increasing with increase in follow up period in patients with LF concentration <280 ng/ml than those with >280 ng/ml (log-rank p < 0.019). Using the cut-off value of 600 ng/ml, the cumulative risk of RP was increasing in patients with <600 ng/ml from day 7 onwards, whereas, there was a slight increase on the cumulative risk of RP on day 14 in patients with LF concentration >600 ng/ml (log-rank p < 0.017) (Fig. 4).

## Discussion

This study describes the prevalence of malaria in pregnancy, its severity and treatment outcome, risk factors for RP and the therapeutic threshold of day 7 LF plasma concentrations that predict treatment outcome in pregnant women from Tanzania. Few studies investigated the LF plasma concentration and treatment outcome in pregnant women previously [13, 22, 23, 38]. The present study, differs from previous studies by describing prevalence of malaria in pregnancy, its severity and risk of RP at three different day 7 plasma LF concentration therapeutic cut-off points, namely <175, <280, and <600 ng/ml to predict malaria treatment failure in pregnant women. The cut-off value of 175 or 280 ng/ml in order to achieve effective therapeutic outcome and 600 ng/ml for maximal efficacy has previously been proposed in studies of non-pregnant adult patients [11, 13]. Altered LF pharmacokinetic properties contributing to the high rates of failure of ALu treatment in later pregnancy is reported previously [14, 22]. Accordingly the suggested plasma LF concentration cut-off points to predict maximal efficacy in non-pregnant adult may not hold true during pregnancy. The present study explored which optimal cut-off value would be pertinent to predict efficacy in malaria infected pregnant women using the proposed reference cut off points for non-pregnant adult patients. In line with the hypothesis of the study and the findings, a recent systematic review indicated low day 7 plasma concentrations is commonly seen in LF studies in pregnant women. This indicates low exposure and possibly reduced efficacy and hence a need for dose optimization to ensure the highest possible efficacy of malaria treatment in pregnant women [21, 22].

Results from this study indicate that 148 (8.1 %) pregnant women were MRDT positive of whom 111 (6.0 %) had parasitologically-proven *P. falciparum* malaria that was confirmed and quantified by microscopy. This figure is lower compared to the prevalence of malaria in pregnancy reported in Lake Zone regions which was 12.2 % [5]. The prevalence of 8.1 % malaria in pregnancy from Tanzania is comparable to other reports in pregnant women from East Africa region such as (9.1 % in Ethiopia [39] and South Sudan [40]), but lower than reports from West Africa (7.7–42 % from Nigeria [41, 42] and 18.1 % from Burkina Faso [43]). Marked decline in the prevalence of malaria in pregnant women from Kenya, as a result of malaria control measures often targeting pregnant women attending ANCs is reported [40]. Baseline epidemiological data from the present study also provides relevant information for policy makers to properly assess the impact of malaria control programme in pregnant women living in Tanzania and sub-Sahara Africa.

The study is well controlled, by excluding data from study participants in whom plasma LF concentration was detected at baseline (day 0). Residual anti-malarial concentrations before treatment in patients with malaria may interfere with outcome of the treatment under investigation and creates bias in drug safety and efficacy assessment [34, 35]. A previous study from Tanzania indicated that more than half (54.1 %) of patients reporting no anti-malarial intake within the last 28 days, had plasma LF concentration above the lower limit of detection [35]. In this study, 7 (8 %) pregnant women had baseline LF concentration >50 ng/mL, indicating drug intake within the last 28 days prior to treatment. Thus data from these patients was excluded during analysis. Based on WHO guidelines on ‘Methods for surveillance of anti-malarial drugs efficacy’, a follow-up of 28 days is recommended as the minimum duration for medicines with elimination half-lives of less than 7 days (including LF) [44]. A longer study follow-up would have increased the risk that more patients (pregnant women) will be lost to follow-up, reducing the study’s validity and sensitivity.

Pharmacokinetics of drugs in pregnancy is altered by several factors, including physiological changes that lower drug absorption, speed up drug clearance, and increase body fluid volume of distribution [28, 29, 45]. In this study, the median day 7 plasma LF concentration (650 ng/mL) found in pregnant women is comparable to previous reports from adult malaria patients in Tanzania (641.4 ng/mL) [46]. The model based prediction of median day 7 LF concentration by Mosha et al. for Tanzanian pregnant (908 ng/mL) and non-pregnant patients (1382 ng/mL) [23] are relatively higher than the findings from pregnant women and other reports for adult population from the same region [46]. The observed high concentrations that have been previously reported within the same region can be due to administration of the anti-malarial drug under direct observed therapy (DOT) [23].

As many as 43.3 % pregnant women did have day 7 LF concentrations of <600 ng/ml which is slightly higher than 31 % which was previously reported in a study conducted by Mosha et al. in Rufiji, Coast region in Tanzania [23]. Moreover, 6.7 and 15 % of pregnant women in this study had day 7 LF plasma concentrations below the previously defined therapeutic cut-offs of 175 ng/ml [11] and 280 ng/ml [19], respectively. These findings are similar to those reported in Uganda but slightly higher to those reported in a previous study in Tanzania [23].

The cure rate in this study was 88.3 %, which is similar to previous report from Rufiji, Tanzania [23]. The median LF concentration on day 7 in patients with RP was significantly lower compared to those with ACPR. Studies have suggested that in order to improve therapeutic efficacy, dose adjustment especially in pregnant women should be taken into consideration to enable a higher day 7 LF concentrations which has been shown to be a marker of treatment outcome [23, 47]. Using population-modeling approach, Mosha et al. predicted lower median venous plasma LF concentrations at day 7 and high treatment failure in pregnant women compared to non-pregnant women [23]. However studies from Uganda reported a minimal alteration of LF pharmacokinetics between pregnant and non-pregnant patients, which was not significant [47, 48]. Thus in the era of emerging drug resistance, further studies with a larger sample size are urgently needed to optimize anti-malarial efficacy in pregnant women, particularly in high transmission regions.

The relative risk of RP increased proportionately when day 7 LF concentration cut-off values increased from <175 to <600 ng/ml. The host immune responses are reduced during pregnancy and this potentially means that a different LF day 7 concentration threshold is required in this special population [26]. The relative risk of RP was 7.8-folds higher with the cut-off point of <600 ng/ml as compared to >600 ng/ml (2.3 and 4.8 at the cut-off points of <175 and 280 ng/ml, respectively) (Fig. 4). Therefore the use of the cut-off points of <175 or < 280 ng/ml may be underestimating the cumulative risk of RP among pregnant women by excluding other patients at risk with LF concentration >280 ng/ml. The findings are in line with previous reports from Thailand where day 7 LF concentration above 600 ng/ml was associated with 100 % efficacy in pregnant women [14].

In areas of high or moderate malaria transmission, response to malaria treatment mainly depends on the host’s immunity, genetic and the amount of drugs available in human plasma to clear the parasites [49, 50]. The study area could be categorized as holoendemic or moderate malaria transmission area. Despite a high number of patients with day 7 LF plasma concentration of <600 ng/ml (45 %), the therapeutic failure rate (11.7 %) in pregnant women in this study was much lower than 16–30 % which was reported in Thailand in 2009 [14, 22]. High cure rates observed in East Africa can be due to the immunity obtained in population living in moderate to high malaria transmission areas [23, 25, 51]. Moreover lower cure rates in Thai pregnant women can be due to higher levels of drug resistance. Prolonged exposure of anti-malarial drugs at sub-therapeutic levels may select for parasites with reduced drug susceptibility and increase the risk for development of drug resistance.

Other baseline characteristics, such as age of the patient, gravida, trimester and baseline parasitaemia were not important factors determining the day 7 LF concentration and subsequent therapeutic response in study participants. This is contrary to what has been reported in a previous study involving pregnant and non-pregnant patients in which patients with higher baseline parasitaemia were more likely to fail treatment [14].

## Conclusions

The findings of this study support that day 7 LF concentrations can be used as a reliable predictor of treatment outcome of malaria in pregnant women. The relative and cumulative risk of RP is higher when using the cut-off point of <600 ng/ml as compared to the previous cut-off points of <175 and <280 ng/ml indicating that even pregnant women with day 7 LF concentrations >280 ng/ml but less than 600 ng/ml are still at high risk of RP. In order to achieve effective therapeutic outcome, higher day 7 venous plasma LF concentration ≥600 ng/ml is required in pregnant patients for maximal efficacy than a cut-off value of 175 or 280 ng/ml, which has been suggested for non-pregnant adult patients. More studies in different areas should be conducted because malaria treatment outcome after ALu administration can be influenced by a number of other factors besides day 7 LF concentration.
